# Supplementary material for: Active suppression prevents the return of threat memory in humans
Source: Commun Biol. 2021 May 21;4:609. doi: 10.1038/s42003-021-02120-2 (PMC8139982; doi:10.1038/s42003-021-02120-2)
Supplement: Supplementary file 2 — Description of Additional Supplementary Files [file 42003_2021_2120_MOESM2_ESM.pdf]

Description of Additional Supplementary Files

File name: Supplementary Data 1

Description: Source data for Fig. 2a.

File name: Supplementary Data 2

Description: Source data for Fig. 2b.

File name: Supplementary Data 3

Description: Source data for Fig. 2c.

File name: Supplementary Data 4

Description: Source data for Fig. 4.
