# Supplementary material for: Active suppression prevents the return of threat memory in humans
Source: Commun Biol. 2021 May 21;4:609. doi: 10.1038/s42003-021-02120-2 (PMC8139982; doi:10.1038/s42003-021-02120-2)
Supplement: Supplementary file 7 — Reporting Summary [file 42003_2021_2120_MOESM7_ESM.pdf]

nature research

Corresponding author(s): Jian Li; COMMSBIO-20-2649B

Last updated by author(s): Jian Li

Reporting Summary

Nature Research wishes to improve the reproducibility of the work that we publish. This form provides structure for consistency and transparency in reporting. For further information on Nature Research policies, see our Editorial Policies and the Editorial Policy Checklist.

Statistics

For all statistical analyses, confirm that the following items are present in the figure legend, table legend, main text, or Methods section.

n/a	Confirmed	
☐	☒	The exact sample size (*n*) for each experimental group/condition, given as a discrete number and unit of measurement
☐	☒	A statement on whether measurements were taken from distinct samples or whether the same sample was measured repeatedly
☐	☒	The statistical test(s) used AND whether they are one- or two-sided<br>*Only common tests should be described solely by name; describe more complex techniques in the Methods section.*
☐	☒	A description of all covariates tested
☐	☒	A description of any assumptions or corrections, such as tests of normality and adjustment for multiple comparisons
☐	☒	A full description of the statistical parameters including central tendency (e.g. means) or other basic estimates (e.g. regression coefficient) AND variation (e.g. standard deviation) or associated estimates of uncertainty (e.g. confidence intervals)
☐	☒	For null hypothesis testing, the test statistic (e.g. F, t, r) with confidence intervals, effect sizes, degrees of freedom and P value noted<br>*Give P values as exact values whenever suitable.*
☒	☐	For Bayesian analysis, information on the choice of priors and Markov chain Monte Carlo settings
☒	☐	For hierarchical and complex designs, identification of the appropriate level for tests and full reporting of outcomes
☐	☒	Estimates of effect sizes (e.g. Cohen's *d*, Pearson's *r*), indicating how they were calculated

Our web collection on statistics for biologists contains articles on many of the points above.

Software and code

Policy information about availability of computer code

Data collection	All skin conductance data were recorded via the Biopac MP160 BioNomadix System.
Data analysis	All skin conductance data analyzed using the Acknowledgement 5.0 software. And IBM SPSS Statistics 23 were used to finish all statistical analysis.

For manuscripts utilizing custom algorithms or software that are central to the research but not yet described in published literature, software must be made available to editors and reviewers. We strongly encourage code deposition in a community repository (e.g. GitHub). See the Nature Research guidelines for submitting code & software for further information.

Data

Policy information about availability of data

All manuscripts must include a data availability statement. This statement should provide the following information, where applicable:

- Accession codes, unique identifiers, or web links for publicly available datasets
- A list of figures that have associated raw data
- A description of any restrictions on data availability

All data of this study is available for download at https://osf.io/qvt6r/.

Field-specific reporting

Behavioural & social sciences study design

All studies must disclose on these points even when the disclosure is negative.

Study description	Quantitative experimental
Research sample	All participants were students recruited from Peking University, Beijing in China. They were right-handed with normal vision and had not participated in electric shock-related experiments before. Our final sample included a total of 55 participants: 28 healthy participants (15 females; mean age = 22.27, SD = 2.64) in experiment 1 and 27 participants (14 females; mean age = 22.12; SD = 2.29) in experiment 2.
Sampling strategy	our sampling procedure is convenience. And we conducted a power analysis 21 (G*Power) to determine the number of participants sufficient to detect a reliable effect. Based on average small-to-medium effect size of reinstatement effect on fear memories reported in the previous literature (median $\eta 2 = 0.19$) , 23 participants for each experiment were needed to detect a significant effect ($\alpha = 0.05$, $\beta = 0.8$, 2 (experiments) x 2 (phases) x 2 (CS) three-way ANOVA interaction effect).
Data collection	All skin conductance data were collected using two Ag-AgCI electrodes attached to the tips of the index and middle fingers of each subject' left hand. Blinding is not possible based on the study design. The experimenter knew when subjects received electrical shocks. The major manipulations were thought suppression and thought diversion,which need experimenter to schedule the subjects for either thought suppression manipulation or thought diversion manipulation.
Timing	Experiment 1 was conducted from September to mid-October 2019. Experiment 2 was conducted December to early January of 2020.
Data exclusions	35 participants (15 females; mean age = 22.4, SD = 2.76) who were either "non-responders" (no SCR response to any CS) or "non-learners" during fear acquisition or extinction were excluded from further analysis: Nineteen participants were excluded after Day 1 of testing for not responding towards non-reinforced CS (CS1+, CS2+ or CS-) (non-responders, n = 9 & 10; Experiment 1 and 2, respectively). Four participants (n = 3 & 1; respectively) failed to show the evidence of fear acquisition since their CS+ responses were numerically smaller than the CS- responses in the latter half trials of acquisition and the difference between the CS+ response and the CS- response also decreased during acquisition. Finally, twelve participants (n = 8 & 4, respectively) failed to show the evidence of fear extinction on day 2, since their CS+ responses were larger than the CS- responses in both the last trial and the latter half trials of extinction and the difference between the CS+ response and the CS- response increased during fear extinction. And the exclusion criteria were pre-established and based on past work.
Non-participation	As these two experiments were two-day studies, some subjects did not return the second day (n=8).
Randomization	We conducted two separate experiments. All participants were randomly allocated to the experiment.

Reporting for specific materials, systems and methods

We require information from authors about some types of materials, experimental systems and methods used in many studies. Here, indicate whether each material, system or method listed is relevant to your study. If you are not sure if a list item applies to your research, read the appropriate section before selecting a response.

Materials & experimental systems

n/a	Involved in the study	
x		Antibodies
x		Eukaryotic cell lines
x		Palaeontology and archaeology
x		Animals and other organisms
	x	Human research participants
x		Clinical data
x		Dual use research of concern

Methods

n/a	Involved in the study	
x		ChIP-seq
x		Flow cytometry
x		MRI-based neuroimaging

Human research participants

Policy information about studies involving human research participants

Population characteristics	All participants were students recruited from Peking University, Beijing in China. They were right-handed with normal vision and had not participated in electric shock-related experiments before. Our final sample included a total of 55 participants: 28 healthy participants (15 females; mean age = 22.27, SD = 2.64) in experiment 1 and 27 participants (14 females; mean age = 22.12; SD = 2.29) in experiment 2.
Recruitment	We recruited subjects by posting experimental information on the Bulletin Board System (BBS) of the Peking university.
Ethics oversight	This study was approved by the ethical committee of Peking University.

Note that full information on the approval of the study protocol must also be provided in the manuscript.
